# Managing Nonagenarians with Acute Myocardial Infarction: Invasive versus Conservative Treatment

**DOI:** 10.1155/2020/8885518

**Published:** 2020-11-10

**Authors:** Jooho Lee, Kyoung-Woo Seo, Jin-Sun Park, Hyoung-Mo Yang, Hong-Seok Lim, Byoung-Joo Choi, So-Yeon Choi, Myeong-Ho Yoon, Gyo-Seung Hwang, Seung-Jae Tahk, Joon-Han Shin

**Affiliations:** ^1^Division of Cardiology, Department of Internal Medicine, Seoul Medical Center, Seoul, Republic of Korea; ^2^Department of Cardiology, Ajou University School of Medicine, Suwon, Republic of Korea

## Abstract

**Background:**

Limited data are available to support an invasive treatment strategy in nonagenarians with acute myocardial infarction (AMI). We aimed to investigate whether percutaneous coronary intervention (PCI) is beneficial in this frail population.

**Methods:**

We retrospectively analyzed 41 nonagenarians with AMI (both ST-segment-elevation and non-ST-segment-elevation MI) between 2006 and 2015 in a single center. We assessed 30-day and one-year mortality rates according to the treatment strategy.

**Results:**

Among study subjects, 24 (59%) were treated with PCI (PCI group) and 17 (41%) were treated with conservative management (medical treatment group) per the clinician's discretion. The median follow-up duration was 30 months (0–74 months). Thirty-day mortality was lower in the PCI group than in the medical treatment group (17% vs. 65%; *P* < 0.001). One-year mortality was also lower in the PCI group than in the medical treatment group (21% vs. 76%; *P* < 0.001). The PCI group presented a 73% decreased risk of death (adjusted hazard ratio: 0.269; 95% confidence interval: 0.126–0.571; *P* < 0.001). In the Killip class 1 through 3 subgroups (*n* = 36), 30-day and one-year mortality were still higher among those in the medical treatment group (13% vs. 54% at 30 days; *P* < 0.001 and 17% vs. 69% at one year; *P* < 0.001). Landmark analysis after 30 days revealed no significant difference in the cumulative mortality rate between the two groups, indicating that the mortality difference was mainly determined within the first 30 days after AMI.

**Conclusion:**

Mortality after AMI was decreased in correlation with the invasive strategy relative to the conservative strategy, even in nonagenarians. Regardless of age, PCI should be considered in AMI patients. However, large-scale randomized controlled trials are needed to support our conclusion.

## 1. Introduction

Better medical developments and improved standards of living have extended the typical life expectancy. More and more aged people are confronting cardiovascular disease, and acute myocardial infarction (AMI) remains one of the leading causes of mortality in this fragile population [1–3]. However, there are limited data available explaining the state of elderly patients with AMI because those aged above 75 to 80 years are underrepresented in most clinical trials [4–10]. Recommended management approaches including percutaneous coronary intervention (PCI) used in younger patients are not proven to be beneficial in this older age group, especially those aged 90 years or older [5, 11, 12]. Some research has suggested the efficacy of an invasive strategy in nonagenarians with AMI [13]. The present study sought to investigate the feasibility and benefit of PCI in comparison with medical treatment in this extremely aged population.

## 2. Methods

### 2.1. Study Population

We retrospectively enrolled 41 patients aged 90years or older admitted for AMI (both ST-segment-elevation MI (STEMI) and non-STEMI) between 2006 and 2015 at Ajou University Hospital (Suwon, Republic of Korea). Among the study subjects, 24 (59%) treated with PCI were assigned to the PCI group and 17 (41%) treated with only medication were assigned to the medical treatment group. All patients underwent follow-up assessments during their in-hospital stay and, after discharge, follow-up data were obtained during patient visits to the outpatient clinic. To confirm patient survival, both the collection of information about insurance cancelation and telephone interviews were used. Per the clinician's discretion, PCI or medical treatment was decided. There were no exclusion criteria for this study. This study was approved by the institutional review board, and because of the retrospective nature of the research, the requirement for informed consent was waived.

### 2.2. Hospital Management

All patients were given loading doses of aspirin 300 mg and clopidogrel 300 to 600 mg upon admission to the emergency room. In the PCI group, the interventional procedure was performed using 6 French guiding catheters via a femoral approach. Unfractionated heparin 100 IU/kg was administered by the intravenous or intracoronary route to achieve an activated clotting time >300 seconds. Type of stent and use of a thrombus aspiration catheter were left up to the attending interventionist's discretion. All patients were admitted to the intensive care unit, at least, 24 hours after hospital admission. In both groups, aspirin 100 mg/d and clopidogrel 75 mg/d were maintained. All other medications including *β*-blockers, angiotensin-converting enzyme inhibitors, statins, nitrates, and diuretics were employed as needed.

### 2.3. Statistical Analysis

All statistical analyses were performed using Statistical Package for the Social Sciences version 20.0 (IBM Corp., Armonk, NY, USA). All values were expressed as mean ± standard deviation (continuous variables) or as number and percentage (categorical variables). The chi-square test was used for categorical variables, and Students *t*-test was used for continuous variables. Kaplan–Meier curves for survival were compared using the log-rank test. Cox proportional-hazards regression analysis was performed to adjust the effects of age, sex, cardiac risk factors, BMI, ejection fraction, renal insufficiency, and Killip class on survival.

## 3. Results

**Figure 1:**
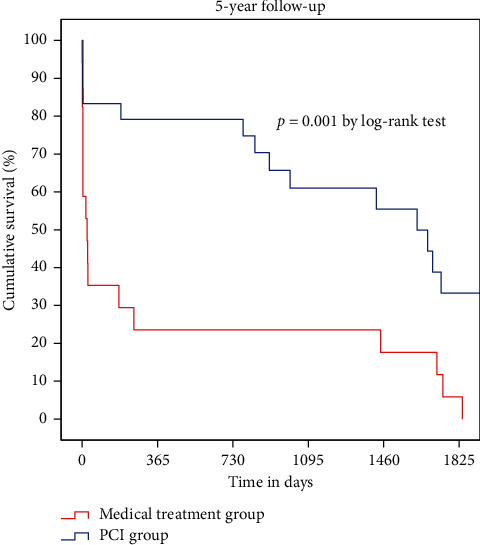
Kaplan–Meier curves of survival in the PCI group and medical treatment group.

**Figure 2:**
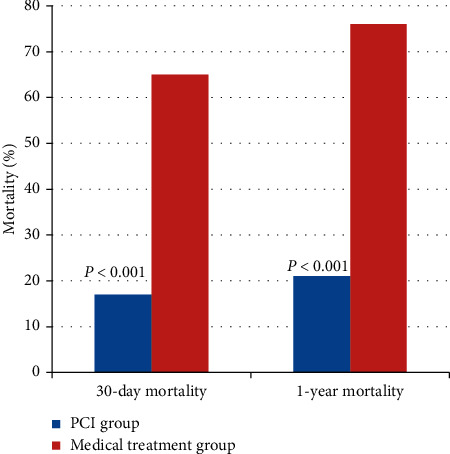
Thirty-day and one-year mortality rates of the PCI group and medical treatment group.

**Figure 3:**
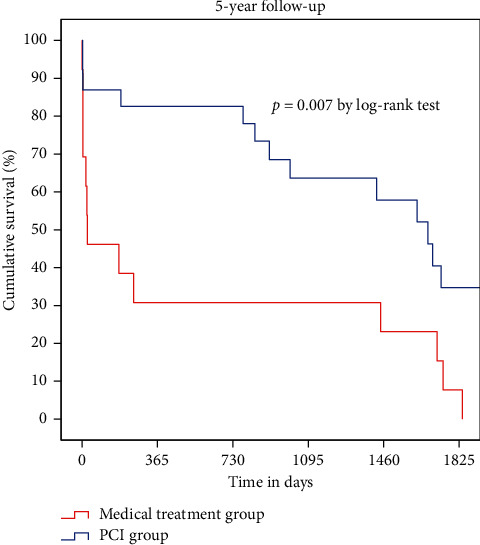
Kaplan–Meier curves of survival in the PCI group and medical treatment group, excluding Killip class 4 patients.

**Figure 4:**
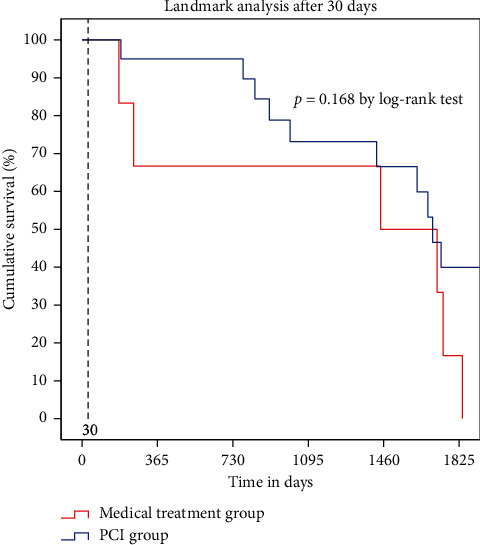
Kaplan–Meier curves of survival in the PCI group and medical treatment group after 30 days.

The median follow-up duration was 30 months (0–74 months), while the mean patient age was 91.3 years (90–99 years), with a female predominance (66%). The baseline demographic and clinical characteristics are summarized in Table 1, while biological parameters and the details of medication logs at discharge are reported in Tables 2 and 3. Age, sex, smoking, diabetes, dyslipidemia, and history of coronary artery disease were not different between the two groups. However, BMI was significantly higher in the PCI group (21.2 vs. 19.0; *P* = 0.02). Kaplan–Meier analysis demonstrated that the PCI group had a higher cumulative survival rate than the medical treatment group (*P* < 0.001 by the log-rank test) (Figure 1). Thirty-day mortality was lower in the PCI group than in the medical treatment group (17% vs. 65%; *P* < 0.001); one-year mortality was similarly lower in the PCI group than in the medical treatment group (21% vs. 76%; *P* < 0.001) (Figure 2). Cox proportional-hazards regression analysis was performed to adjust the effects of age, sex, cardiac risk factors, BMI, ejection fraction, renal insufficiency, and Killip class on death (Table 4). The PCI group had a 73% decreased risk of death (adjusted hazard ratio: 0.269; 95% confidence interval: 0.126–0.571; *P* < 0.001). In the subgroup containing those with Killip classes 1 through 3 (*n* = 36), 30-day and one-year mortality were still higher in the medical treatment population (13% vs. 54% at 30-days; *P* < 0.001 and 17% vs. 69% at one year; *P* < 0.001) (Figure 3). According to landmark analysis after 30 -days, there was no significant difference in the cumulative mortality rate between the two groups, indicating that the mortality difference was predominantly established within the first 30 days after AMI (*P* = 0.168 by the log-rank test) (Figure 4).

## 4. Discussion

The number of very elderly patients with AMI is rapidly growing because the typical life expectancy has increased. In general, treatment results of elderly patients are expected to be worse because of their different clinical profile relative to that of younger patients [9]. A delay in visiting the hospital is common among older patients due to the presence of atypical or vague symptoms, diminished pain sensation, cognitive impairment, and social constraints. Various comorbidities and low physical strength also are obstacles to a conventional invasive treatment approach [11–14]. However, the clinical outcomes of these aged patients (>75 years) have been improving over the last 15 years due to appropriate changes in early procedural and medical management according to data from French registries [1]. No age limitation has been stated in the guidelines for reperfusion management of STEMI patients from the American College of Cardiology Foundation/American Heart Association since 2013 [15]. However, in extremely aged AMI patients older than 90 years, such data are scarce. In most clinical trials, nonagenarians with AMI are underrepresented despite the increasing incidence and risk of AMI among them. The risks and benefits associated with PCI among nonagenarians with AMI are not well understood. Thus, many older patients are still undertreated conservatively in real-world practice regardless of PCI feasibility. [1, 3, 5, 12, 13, 16–20].

Previous studies including registry data for nonagenarians with AMI emphasized the outcomes of PCI without involving a control group. As such, the primary strength of our study is the presence of a control group. To the best of our knowledge, our study is the first to compare the short-term and long-term outcomes of PCI to medical treatment only among nonagenarian AMI patients. Our results suggested that 30-day and one-year mortality were much lower in the PCI group than in the medical treatment group, indicating that deploying an invasive approach in nonagenarians with AMI is reasonable (17% vs. 65% for 30 days and 21% vs. 76% for one year). Previous studies involving nonagenarian AMI patients treated with PCI presented similar results [21]. Helft et al. reported a 24.9% mortality rate in a cohort of 418 nonagenarians with STEMI undergoing hospital follow-up only. [22] Petroni et al. reported a 24% hospital mortality rate and a 47% one-year mortality rate among 145 nonagenarians with STEMI [4]. However, in our study, treatment strategy was not decided by randomization, and the percentage of Killip class 4 patients was higher in the medical treatment group, meaning that hemodynamically unstable patients were prone to be treated conservatively per the clinician's discretion. The mortality benefit might come from treating more stable patients. Cox proportional-hazards regression analysis was used to adjust for the effects of age, sex, cardiac risk factors, BMI, ejection fraction, and Killip class on death. After adjustment, the PCI group showed about three times the survival benefit of the medical treatment group. Also, the subgroup analysis, which excluded Killip class 4 patients, showed a consistent mortality benefit for the PCI group (13% vs. 54% at 30 days and 17% vs. 69% at one year). In patients who were discharged alive in the study of Petroni et al., the one-year mortality rate was very low. [4] Landmark analysis after 30 days showed no significant difference in cumulative mortality rate, suggesting that the difference in mortality was generally determined within the first 30 days after AMI. In other words, in-hospital management with PCI has contributed to a survival benefit over medical management. According to data from the Korean Statistical Information Service, the age-specific annual death rate affecting those between the ages of 90 and 94 years in the Korean general population was 17.5% (21% in men and 16.5% in women). The one-year mortality rate in the PCI group did not exceed that for the same age group in the general population, meaning that aggressive intervention was not significantly more harmful in this vulnerable group.

In terms of bleeding risk, substantial portion of elderly patients are at high bleeding risk. The age above 75 is placed in the minor criteria for high bleeding risk at the time of PCI. In the present study, more than half of enrolled patients were categorized into high bleeding risk according to the Academic Research Consortium for High Bleeding Risk (ARC-HBR) criteria [23]. However, full hemocompatibility of metallic stent has not yet been reached, and many factors related to high bleeding risk also contribute to increase ischemic complication [24]. Study patients maintained dual antiplatelet, at least, 1 year if not dead. Further investigation is needed to confirm that how many portion of bleeding complication contribute to mortality in these elderly AMI patients on dual antiplatelet.

The present study has several limitations that must be kept in mind. First, it was a retrospective, nonrandomized, single-center experience with a small study cohort. Adequate recruitment of nonagenarians with AMI is very difficult, and randomization may provoke ethical problems. The PCI group may be highly selective and relatively more stable than the medical treatment group. We cannot make a definite conclusion about the absolute benefits of PCI. Second, causes of death were not specified because we had no options but to conduct telephone interviews and gather additional insurance cancelation information to investigate adverse clinical events. Cardiovascular mortality was not available in the present study. Third, we did not provide detailed clinical and procedural findings due to a lack of data. Time onset to presentation and angiographic severity may be important factors affecting the treatment results.

## 5. Conclusions

This study suggests that short-term and long-term outcomes were better among nonagenarians with AMI after an invasive strategy rather than a conservative one. It is reasonable to comply with contemporary recommendations from the data collected from younger patients regardless of age in clinical practice. However, large-scale randomized controlled trials are needed to support this suggestion.

## Figures and Tables

**Table 1:** Baseline demographic and clinical characteristics.

	PCI group (*n* = 24, 59%)	Medical treatment group (*n* = 17, 41%)	*P* value
Age (years)	90.8 ± 1.6	91.9 ± 2.3	0.073
Male sex, *n* (%)	9 (38)	5 (29)	0.591
Body mass index (kg/m^2^)	21.2 ± 2.8	19.0 ± 3.2	0.023
Hypertension, *n* (%)	14 (58)	10 (59)	0.975
Diabetes, *n* (%)	5 (21)	3 (18)	0.800
Dyslipidemia, *n* (%)	3 (13)	1 (6)	0.482
Smoking, *n* (%)	5 (21)	3 (18)	0.800
History of coronary artery disease, *n* (%)	4 (17)	2 (12)	0.662

Killip class at admission, *n* (%)			
I	12 (50)	8 (47)	0.853
II	5 (21)	3 (17)	0.800
III	6 (25)	2 (12)	0.292
IV	1 (4)	4 (24)	0.062
LVEF at admission (%)	43 ± 14	45 ± 14	0.694
High bleeding risk by ARC-HBR criteria, *n* (%)	17 (71)	11 (64)	0.678

LVEF, left ventricular ejection fraction; ARC-HBR, the Academic Research Consortium for High Bleeding Risk.

**Table 2:** Biological parameters.

	PCI group	Medical treatment group	*P* value
Cardiac TnI peak (ng/mL)	12.5 ± 17.7	10.2 ± 12.6	0.641
CK-MB (ng/mL)	116.6 ± 132.5	140.7 ± 169.6	0.611
Serum creatinine (mg/dL)	1.4 ± 0.6	1.2 ± 0.3	0.242
Total cholesterol (mg/dL)	148 ± 41	144 ± 40	0.741
LDL-C (mg/dL)	94 ± 37	85 ± 35	0.437
HDL-C (mg/dL)	43 ± 13	45 ± 13	0.522
Triglycerides (mg/dL)	60 ± 27	65 ± 39	0.635

TnI, troponin I; CK-MB, creatinine kinase myocardial band; LDL-C, low-density lipoprotein cholesterol; HDL-C, high-density lipoprotein cholesterol.

**Table 3:** Medication logs at discharge.

	PCI group	Medical treatment group	*P* value
Aspirin, *n* (%)	20 (100)	10 (100)	
Clopidogrel, *n* (%)	20 (100)	9 (90)	0.150
Beta-blockers, *n* (%)	20 (100)	9 (90)	0.150
Statins, *n* (%)	20 (100)	10 (100)	
ACE inhibitors or ARBs, *n* (%)	19 (95)	7 (70)	0.058

ACE, angiotensin-converting enzyme; ARBs, angiotensin receptor blockers. Cases of in-hospital mortality were excluded.

**Table 4:** Results of multivariate Cox regression model.

Covariate	Coefficient	Standard error	*P* value	Hazard ratio	95% CI lower	Upper
Age	0.117	0.099	0.236	1.124	0.926	1.363
Sex	−0.575	0.622	0.355	0.563	0.166	1.903
BMI	−0.064	0.069	0.356	0.938	0.819	1.075
Hypertension	−0.187	0.511	0.714	0.829	0.305	2.256
Diabetes	−0.331	0.541	0.541	0.718	0.249	2.074
Dyslipidemia	−0.120	1.279	0.925	0.887	0.072	10.875
Smoking	−0.247	0.723	0.732	0.781	0.179	3.224
History of CAD	0.035	0.673	0.958	1.036	0.277	3.873
Killip class	0.708	0.180	≤0.001	2.029	1.425	2.890
PCI	−1.315	0.385	0.001	0.269	0.126	0.571

CI, confidence interval; BMI, body mass index; CAD, coronary artery disease; PCI, percutaneous coronary intervention.

## Data Availability

The datasets used are available from the corresponding author upon reasonable request.
